# Distinguishing Relational Aspects of Character Strengths with Subjective and Psychological Well-being

**DOI:** 10.3389/fpsyg.2017.01159

**Published:** 2017-07-11

**Authors:** Melanie Hausler, Cornelia Strecker, Alexandra Huber, Mirjam Brenner, Thomas Höge, Stefan Höfer

**Affiliations:** ^1^Department of Medical Psychology, Medical University of Innsbruck Innsbruck, Austria; ^2^Institute of Psychology, University of Innsbruck Innsbruck, Austria

**Keywords:** well-being, character strengths, virtues, medical students, positive psychology, interventions

## Abstract

Research has shown that character strengths are positively linked with well-being in general. However, there has not been a fine-grained analysis up to date. This study examines the individual relational aspects between the 24 character strengths, subjective well-being (SWB), and different aspects of psychological well-being (PWB) at two times of measurement (*N* = 117). Results showed that overall the “good character” was significantly stronger related with PWB than with SWB. The character strength “hope” was at least moderately correlated with the PWB aspects meaning, optimism and autonomy, and “zest” with the PWB aspects relationships and engagement. “Persistence” showed the highest correlation with the PWB aspect mastery. Out of the 24 character strengths, the happiness-related strengths (hope, zest, gratitude, curiosity, and love) were more likely to correlate with PWB and SWB than any other character strength. This study offers a more fine-grained and thorough understanding of specific relational aspects between the 24 character strengths and a broad range of well-being aspects. Future studies should take up a detailed strategy when exploring relationships between character strengths and well-being.

## Introduction

Virtues, character strengths and their relationship to well-being were first highlighted by Peterson and Seligman in 2004. In the following years, an increasing number of scientific publications appeared in print in this field. Different types and clusters of well-being were analyzed and positively linked with virtues and character strengths on a theoretical (e.g., Seligman, 2011) and an empirical basis (e.g., Proyer et al., 2011). Scholars distinguished two forms of well-being: subjective well-being (SWB) (i.e., the pleasant life) and psychological well-being (PWB) (i.e., the life of values) (Keyes et al., 2002; Deci and Ryan, 2008; definitions see below). Intervention studies showed that SWB and PWB can be increased by fostering character strengths (Quinlan et al., 2012). These results highlight that character strengths are a potential starting point for increasing individual well-being in general. Several studies have indicated that certain character strengths are more beneficial for well-being in general than others (Park et al., 2004). But until now, the complex relations between different character strengths and different aspects of well-being have not been explored systematically. A recent review showed that most studies used life satisfaction as an indicator of (subjective) well-being, whereas PWB and its sub-dimensions were rarely studied (Quinlan et al., 2012). There has been no comprehensive study analyzing different aspects of SWB and PWB and their relations with character strengths until now.

Research results show, that medical students (56%), resident physicians (60%) and physicians (51%) have a high prevalence of burnout compared to the general population (Dyrbye et al., 2014). A representative study in the USA showed a higher likelihood of depression (controlled for gender, age, relationship and career status) for medical students compared to the general population and to other courses of studies (Brazeau et al., 2014). Reports of burnout, distress and depression in samples of German-speaking medical students correspond to these findings in the US (Voltmer et al., 2012). As a consequence, Dyrbye et al. (2013) emphasized that efforts should be made to prevent burnout and foster career satisfaction from the beginning of medical studies. Basic research should therefore aim for a more detailed understanding of well-being and character strengths in this context. Against this background, the aim of this study was to examine the different relations between various aspects of well-being and character strengths in a sample of medical students at two points in time.

### Subjective and psychological well-being

SWB comprises, on the one hand, the cognitive component of satisfaction with life as a whole, and on the other hand the presence of positive emotions (e.g., joy, interest, love, or pride) and the absence of negative emotions (e.g., loneliness, anger, anxiety) as affective components (Seligman, 2002; Fredrickson, 2009). PWB consists of autonomy, environmental mastery, personal growth, personal relationships, purpose in life, and self-acceptance (Keyes et al., 2002). SWB and PWB are related but empirically distinct (Keyes et al., 2002; Ring et al., 2007).

Su et al. (2014) recently reviewed, compared and integrated existing prominent well-being theories. They identified six aspects of PWB alongside SWB. These are key indicators based on established well-being theories, namely self-determination theory by Ryan and Deci (2000) and Ryff's (1995) theory of psychological well-being, and Seligman's (2011) PERMA well-being model. Building on these theories Su et al. (2014) developed a well-being questionnaire *(Comprehensive Inventory of Thriving)* measuring SWB and the following six theoretical based aspects of PWB:

*Relationships:* One of the three psychological basic needs postulated in the self-determination theory (Ryan and Deci, 2000) is the need for relatedness (besides competence and autonomy). Ryff (1995), Seligman (2011), and Diener et al. (2009) demonstrated the importance of positive relationships for different well-being concepts. Relationships include mutual support, community, trust, respect, belonging and the opposite of loneliness (Su et al., 2014).
*Engagement:* The engaged life is one of the three routes to happiness postulated by Seligman in 2002. Being engaged, knowing one's strengths and using them, contributes to psychological well-being, flourishing and flow experiences (Peterson and Seligman, 2004).
*Meaning:* The meaningful life is another route to happiness (Seligman, 2002). Ryff (1995) postulated that purpose in life is an essential part of PWB. If people know what gives meaning and purpose to their lives and act accordingly, their level of happiness increases.
*Mastery:* Ryff (1995) labeled this dimension environmental mastery, Ryan and Deci (2000) competence; in Seligman's PERMA model, it is the factor of accomplishment (Seligman, 2011). Diener included the factors of self-esteem and mastery in his concept of flourishing (Pearlin and Schooler, 1978; Diener et al., 2009), focused on generalized self-efficacy and Judge et al. (2003) referred to self-esteem, self-efficacy and locus of control in their concept of core self-evaluations. All these concepts have guided research showing that feeling competent in managing (or mastering) daily tasks or challenges (= the environment) promotes well-being. Mastery includes skills, learning, accomplishment, self-efficacy, and self-worth (Su et al., 2014).
*Autonomy:* Autonomy is one of the basic needs (Ryan and Deci, 2000). Feeling autonomous and having control of one's life is an essential part of human well-being (Pearlin and Schooler, 1978).
*Optimism:* Optimism is defined as expecting positive things in life and thinking optimistically about the future instead of having pessimistic expectations (Pearlin and Schooler, 1978; Scheier and Carver, 1985; Diener et al., 2009).

### Character strengths and well-being

Peterson and Seligman (2004) postulated the so-called “good character” to be essential for human functioning. Moreover, character strengths and their expression differ between individuals and make people unique. Peterson and Seligman (2004) identified 24 individual character strengths (e.g., bravery, humor, curiosity) which were assigned to six historically based human virtues, namely courage, justice, humanity, temperance, transcendence, and wisdom. The classification of character strengths are presented in the *Values in Action Inventory of Strengths* (VIA-IS) and 10 criteria were set which define character strengths in general (Peterson and Seligman, 2004). According to these criteria, character strengths are universally valid, contribute to the “good life” in several ways and are fulfilling, morally valued in themselves, do not evoke envy, have a negative counterpart, and constitute individual differences that are trait-like, measurable, unique and distinct from the other character strengths, have paragons and prodigies, can be absent in some people, and can be fostered by institutions and rituals. Peterson and Seligman (2004) argued that every person has three to seven character strengths which are especially important for the individual. These strengths are called “signature strengths” and are defined as central strengths for the individual which fulfill several criteria such as “a sense of ownership and authenticity,” “a feeling of excitement while displaying it (…),” or “intrinsic motivation to use the strength” (Peterson and Seligman, 2004). Short descriptions of the 24 character strengths are provided in online Supplementary Material.

Peterson and Seligman assumed that character strengths underpin well-being and lead to more positive emotions, engagement, meaning, accomplishment, and better relationships (Peterson and Seligman, 2004; Seligman, 2011). The empirical research also found character strengths in general to contribute to subjective well-being, mental and physical health and satisfaction with life as well as work satisfaction (e.g., Peterson and Park, 2011; Allan and Duffy, 2014; Douglass and Duffy, 2014). A positive relation of the “good character” with life satisfaction was identified in several studies (e.g., Ruch et al., 2007; Martínez-Martí and Ruch, 2014) in German speaking countries. Moreover, the 24 character strengths were analyzed in more detail in relation to SWB. Many studies have identified hope, zest, gratitude, curiosity, and love as the five character strengths most strongly related with life satisfaction (e.g., Park et al., 2004; Buschor et al., 2013). Littman-Ovadia et al. (2017) called these five strengths the “happiness strengths.” Park et al. (2004) further identified the five character strengths least related to life satisfaction: modesty, creativity, appreciation of beauty and excellence, judgment, and love of learning. Some studies found transcendence strengths in general (appreciation of beauty and excellence, gratitude, hope, humor, and spirituality) to be the strongest predictors of life satisfaction (Shoshani and Slone, 2013; Weber et al., 2013) and positive affect (Weber et al., 2013). In addition to replicating the highest correlations between life satisfaction and the happiness strengths, Proyer et al. (2011) found hope and spirituality to be the best predictors of future life satisfaction. Peterson et al. (2007) examined relationships between character strengths and the three routes to happiness: meaning, engagement and pleasure. They found that religiousness, gratitude, hope, zest, and curiosity were the character strengths associated with meaning, the character strengths of zest, curiosity, hope, perseverance, and perspective were strongly correlated with engagement, and humor, zest, hope, social intelligence, and love with the SWB aspect of pleasure. Some longitudinal studies have also focused on the link between character strengths and SWB, and shown the potential of fostering well-being through character strengths interventions (Proyer et al., 2013).

Overall most studies have focused on SWB (especially on life satisfaction) as a general well-being indicator, and less on PWB aspects (such as meaning or engagement) when exploring their relationships with character strengths. The results indicate that specific character strengths are more important than others (e.g., hope, zest, gratitude, curiosity, and love) and different strengths are important for different aspects of SWB (e.g., Weber et al., 2013). Results are not definite and vary (e.g., due to sampling issues) with respect to the importance of character strengths.

### Aims of the present study

The purpose of the study was a more fine-grained analysis exploring the associations of the specific 24 character strengths with the distinct aspects of SWB and PWB in a sample of medical students. Fine-grained analyses are indicated due to the following reasons:

Firstly, more detailed understanding of relational aspects of character strengths and distinct well-being aspects is relevant for tailoring character strengths interventions. According to specific well-being aspects an individual may want to improve, an intervention can focus at a particular character strength which is most related to that particular well-being aspect. In the long term this can become a basis for guidelines for practitioners as well as for individuals.

Secondly, if there are different relationships between the 24 character strengths and well-being aspects it is important for future studies to take up a detailed strategy when exploring relationships between character strengths and well-being.

Thirdly, a more fine-grained analysis increases the comparability of research results. Future studies will need to take specific wording into account for a more precise, clear and sustainable research practice in relation to character strengths and different aspects of SWB and PWB.

Finally, we are not aware of any study analyzing relational aspects of character strengths and well-being in the context of medical students. This analysis will give a first basis for future character strengths intervention studies for the well-being of medical students.

In this study, we identified four main research questions examining data of medical students in their first year of study (t1) and one year later (t2).

(1) Which character strengths show the strongest associations with SWB?
According to the literature (e.g., Park et al., 2004; Buschor et al., 2013) we hypothesized that out of all character strengths, the happiness strengths of hope, zest, gratitude, curiosity and love at t1 would show the highest correlations with SWB at t1 and one year after at t2 (hypothesis 1).
(2) Which character strengths are most strongly associated with PWB?
Again, we hypothesized that the happiness strengths at t1 would be most strongly correlated with PWB at t1 and t2 compared to the other character strengths (hypothesis 2).
(3) Does “good character” in general have a stronger association with PWB than with SWB?
Individual character strengths are part of living the “good life” and differ from positive subjective experiences and emotions (SWB; Seligman and Csikszentmihalyi, 2000). It was therefore hypothesized that the “good character” at t1 shows significantly higher correlations with PWB than with SWB at t1 and t2 (hypothesis 3).
(4) How are the character strengths related to psychological well-being aspects?
This question was examined by taking an exploratory look at the relationships between the 24 character strengths and the six scores of PWB at t1 and t2. Our aim was to identify the character strengths most highly correlated with the specific well-being aspects in order to provide tentative indications and recommendations for future research focusing on medical students. This is the first study to analyze relationships between character strengths and a specific spectrum of well-being aspects in the context of medical education at two points in time.

## Methods

### Study design and sample

The Board for Ethical Questions in Science of the University of Innsbruck provided approval for the study. Medical students participated in an online survey twice, with measuring times a year apart (t1: January/February 2015 with *N* = 178 and t2: January/February 2016 with *N* = 121). We offered direct feedback on participants' personal five signature strengths, medical education credits, and a raffle of medical books relevant to their current semester. We collected the data at a medical university in Austria as a part of a larger research project. Complete longitudinal data was available for *N* = 117 [66% female, mean age 20.3 ± 2.0 years (t1) and 21.4 ± 2.1 (t2) respectively]. Most were Austrian nationals (56%), German (23%), or Italian (20%); 2% had other nationalities. 74/50% (t1/t2) were single. 24/22% (t1/t2) were living alone, 11/12% (t1/t2) with a partner, 48/54% (t1/t2) in a flat share and 17/12% (t1/t2) with their parents or family of origin. Participants spent an average of 37.5 ± 15.4 (t1) and 35.6 ± 13.8 (t2) h per week studying. 17/26% (t1/t2) reported secondary employment with an average of 8.8 ± 4.0 (t1) and 9 ± 4.8 (t2) h per week.

### Measures

#### Well-being

The valid and reliable German version of the *Comprehensive Inventory of Thriving* (CIT; Su et al., 2014; Hausler et al., 2017) was used to measure well-being. It comprises 54 items rated on a five-point Likert scale ranging from *strongly disagree* (= 1) to *strongly agree* (= 5). The 54 items measure 18 aspects of SWB and PWB (3 items for each aspect). Three of the 18 aspects are parts of SWB (life satisfaction, negative and positive emotions). The other 15 aspects are parts of PWB and are further summarized into six different components: relationships, engagement, meaning in life, mastery, autonomy, and optimism. Different well-being scores can be calculated based on detailed confirmatory factor analyses, including higher-order factors (Hausler et al., 2017):

1. A total mean score serves as a measure of general well-being (= *Thriving*).
2. Scales for SWB and PWB can be calculated separately.
3. Each of the six PWB subscales can be interpreted on its own.
4. Each of the 18 well-being subscales can be interpreted on its own.

We calculated PWB and SWB scores separately (option 2) to address hypotheses 1–3. To analyze PWB in more detail (research question 4), we also calculated the six PWB subscales (relationships, engagement, meaning in life, mastery, autonomy, optimism; option 3). In order to depict the relations in a compact and concise form, we did not additionally report on the 18 well-being subscales (option 4) and focused on the umbrella terms. The general well-being score (option 1) was not used either since our focus was on the specific well-being aspects. The reliabilities (Cronbach's Alphas) in the German validation study (Hausler et al., 2017) were as follows: relationships (α = 0.87), engagement (α = 0.83), mastery (α = 0.90), autonomy (α = 0.78), meaning (α = 0.82), optimism (α = 0.87), SWB (α = 0.95), and PWB (α = 0.94). The reliabilities of the scales in the present study were at least acceptable for all CIT scales ranging from excellent values of α = 0.94/0.94 (t1/t2: SWB) to α = 0.75/0.85 (t1/t2: autonomy) (Table 1).

**Table 1:** Reliability, comparison of means and intercorrelations of character strengths at t1 and t2.

**Variables**	**Means (SD)**		***p*-values^#^**	**Inter-correlations^§^**	**α (t1/t2)**
	**t1**	**t2**			
**(1) WISDOM AND KNOWLEDGE**					
Creativity	3.36 (0.73)	3.38 (0.70)	0.74	0.75	0.85/0.87
Curiosity	3.81 (0.59)	3.85 (0.61)	0.36	0.68	0.70/0.79
Open-mindedness	4.05 (0.69)	4.09 (0.60)	0.31	0.79	0.83/0.80
Love of learning	3.47 (0.70)	3.70 (0.63)	**<0.001**	0.70	0.67/0.64
Perspective	3.63 (0.65)	3.70 (0.64)	**0.043**	0.69	0.76/0.79
**(2) COURAGE**					
Authenticity	4.30 (0.46)	4.30 (0.50)	0.90	0.56	0.66/0.71
Bravery	3.52 (0.71)	3.58 (0.64)	0.228	0.73	0.76/0.76
Persistence	3.86 (0.62)	3.89 (0.65)	0.44	0.72	0.72/0.80
Zest	3.65 (0.64)	3.66 (0.68)	0.91	0.71	0.76/0.79
**(3) HUMANITY**					
Kindness	4.30 (0.50)	4.29 (0.49)	0.69	0.64	0.72/0.76
Love	4.04 (0.62)	4.03 (0.64)	0.83	0.68	0.67/0.72
Social intelligence	4.00 (0.51)	3.98 (0.51)	0.61	0.64	0.66/0.66
**(4) JUSTICE**					
Fairness	4.13 (0.56)	4.14 (0.51)	0.72	0.64	0.72/0.72
Leadership	3.65 (0.56)	3.71 (0.58)	0.149	0.66	0.65/0.72
Teamwork	3.65 (0.57)	3.69 (0.55)	0.44	0.64	0.71/0.69
**(5) TEMPERANCE**					
Forgiveness	3.64 (0.67)	3.61 (0.58)	0.59	0.62	0.69/0.66
Modesty	3.39 (0.59)	3.41 (0.58)	0.70	0.66	0.57/0.61
Prudence	3.63 (0.67)	3.68 (0.05)	0.37	0.65	0.73/0.68
Self-regulation	3.29 (0.73)	3.30 (0.73)	0.74	0.71	0.64/0.68
**(6) TRANSCENDENCE**					
Appreciation of beauty and excellence	3.56 (0.77)	3.58 (0.67)	0.74	0.72	0.78/0.65
Gratitude	3.75 (0.59)	3.70 (0.63)	0.251	0.67	0.70/0.76
Hope	3.79 (0.71)	3.78 (0.71)	0.90	0.63	0.70/0.75
Humor	3.85 (0.64)	3.80 (0.66)	0.233	0.73	0.78/0.82
Spirituality	2.42 (1.11)	2.39 (1.12)	0.59	0.86	0.92/0.92
**(7) VIA TOTAL SCORE**	3.70 (0.32)	3.72 (0.33)	0.34	0.73	0.86/0.89
**(8) WELL-BEING ASPECTS**					
SWB	4.02 (0.68)	3.96 (0.70)	0.29	0.58	0.94/0.94
PWB	4.02 (0.39)	3.96 (0.68)	0.103	0.75	0.91/0.93
Relationships	3.88 (0.46)	3.81 (0.49)	**0.037**	0.71	0.84/0.85
Engagement	3.90 (0.68)	3.98 (0.65)	0.129	0.64	0.80/0.85
Mastery	4.10 (0.48)	4.05 (0.50)	0.28	0.54	0.88/0.89
Autonomy	4.35 (0.76)	4.33 (0.75)	0.78	0.32	0.75/0.85
Meaning	3.95 (0.92)	3.95 (0.89)	1.00	0.68	0.85/0.83
Optimism	4.18 (0.67)	4.10 (0.72)	0.165	0.63	0.79/0.83

N = 117; t1, measurement time 1; t2, measurement time 2;

# p values of mean difference;

§ intercorrelations between t1 and t2; bold p values indicate statistically significant differences between the means of t1 and t2; ^**^all intercorrelations were significant with p < 0.010.

#### Character strengths

To measure individual character strengths, the German 120-item version of the *Values in Action Inventory of Strengths (VIA-120)* (Institute on Character, 2014) was used. Items are rated from *strongly agree* (= 5) to *strongly disagree* (= 1) on a five-point scale. The validation of the VIA-120 shows, that “the brief version is substantially equivalent to the original long version in internal reliability and validity” (Littman-Ovadia, 2015, p. 236). In the present study the psychometric properties were very similar to the original 240-item version, with Cronbach's alpha ranging from α = 0.57/0.61 (t1/t2: modesty) to α = 0.92 (t1/t2: spirituality) for the short version (Littman-Ovadia, 2015). The reliability of the VIA-120 total score (mean across all 24 subscales) was good, at α = 0.86/0.89 (t1/t2) (Table 1).

### Data analysis

We analyzed data at the level of specific character strengths. First, we computed descriptive statistics and internal consistencies (Cronbach's Alphas) of the measures. Second, we compared means of measurement at time 1 and 2 (paired sample *t*-test and Cohen's d) and analyzed intercorrelations of the means at t1 and t2. Pearson's correlations coefficients were interpreted as follows: *r* of < 0.10 = no effect, *r* = 0.10–0.29 = weak effect, *r* = 0.30–0.49 = moderate effect, *r* ≥ 50 = high effect. Third, we calculated correlations with demographics (age, sex, family status), as proposed in several studies (e.g., Harzer and Ruch, 2012; Wagner and Ruch, 2015). All subsequent analyses were controlled for demographics. To test hypotheses 1, 2, and 3, we computed partial correlations between character strengths and well-being aspects. We conducted Steiger tests (one-tailed) for comparing dependent correlations (Steiger, 1980).

In order to analyze the specific relationships between the well-being aspects and each of the 24 character strengths the data was analyzed as follows: (1) partial correlations between all character strengths and the six PWB aspects were computed; (2) hierarchical multiple regressions were calculated, including only significantly correlated character strengths identified by the partial correlations (first step: demographics, method: enter; second step: significantly correlated character strengths, method: stepwise).

## Results

### Preliminary analyses

#### Descriptives, intercorrelations, and comparison of the means

CIT scores and the VIA-120 total score were normally distributed at t1 and t2 (KS-test, histogram) and no outliers were present (critical *z*-value of 3.29 of CIT and VIA-120 scores, boxplot). In order to analyze collinearity, we correlated all character strengths at t1 with each other. The highest intercorrelations were found between curiosity and zest (*r* = 0.71), hope and zest (*r* = 0.69), fairness and leadership (*r* = 0.63), teamwork and leadership (*r* = 0.61), and gratitude and hope (*r* = 0.59). Additionally, we compared the means in order to identify the highest-ranked strengths. Results show that the medical students had the highest means in the strengths of authenticity, kindness, and fairness at t1 and t2 (Table 1). In order to analyze stability we compared the means of all variables between t1 and t2. We found no statistical differences between t1 and t2, except for a significant increase in the character strengths of perspective and love of learning, with a very small effect size of *d* = 0.07 for perspective, and moderate effect size of *d* = 0.44 for love of learning. No well-being aspects changed significantly between t1 and t2, except for a slight decrease of PWB-relationships with a small effect size of *d* = 0.20 (Table 1). Additionally, to analyze stability at the level of the individual, we intercorrelated all variables of t1 with their counterparts at t2. Correlation coefficients were high and, without exception, statistically significant (*p* < 0.001). Intercorrelations of character strengths at t1 and t2 ranged from *r* = 0.56 (authenticity) to *r* = 0.86 (spirituality), and those of well-being aspects from *r* = 0.32 (autonomy) to *r* = 0.75 (PWB). To address demographics as confounding variables, we investigated possible effects of sex, age, and family status (single or partner relationship). Singles showed a lower score in love (t1/t2); age was positively associated with love of learning (t1) and humor (t1), and negatively with gratitude and spirituality. Men reported higher curiosity (t2), open-mindedness (t1/t2), wisdom (t1/t2), prudence (t1/t2), and self-regulation (t1). Based on these results, subsequent analyses were controlled for demographics.

### Which character strengths show the strongest associations with SWB?

We had hypothesized that hope, zest, gratitude, curiosity and love at t1 would be correlated most strongly with SWB at t1 or t2 (hypothesis 1). Results mostly confirmed hypothesis 1 at t1 and t2 for hope (*r* = 0.67/0.41), zest (*r* = 0.51/0.42), gratitude (*r* = 0.45/0.27), curiosity (*r* = 0.39/0.34), and love (*r* = 0.41/0.21). The character strengths of humor at t1 (*r* = 0.30) and spirituality at t2 (*r* = 0.31) were also moderately correlated with SWB. According to the Steiger tests the correlation of SWB and humor (t1) was only significantly different compared to the two highest correlations of SWB with zest (*p* = 0.003) and hope (*p* < 0.001). The correlation of SWB and spirituality (t2) was not statistically different to all of the happiness strengths correlations. All other character strengths had no significant or only small (*r* < 0.30) correlations with SWB (Table 2).

**Table 2:** Partial correlations between character strengths measured at time 1 (t1) and well-being measured at t1 and time 2 (t2).

**Character strengths at t1**	**SWB t1/t2**	**PWB t1/t2**	**Relationships t1/t2**	**Engagement t1/t2**	**Meaning t1/t2**	**Mastery t1/t2**	**Autonomy t1/t2**	**Optimism t1/t2**
**(1) WISDOM AND KNOWLEDGE**								
Creativity	0.04/−0.05	0.28^**^/0.09	0.10/0.08	0.29^**^/0.10	0.14/0.04	0.41^**^/0.16	0.09/−0.08	0.05/−0.12
Curiosity	0.39^**^/0.34^**^	0.52^**^/0.48^**^	0.30^**^/0.37^**^	0.49^**^/0.53^**^	0.38^**^/0.34^**^	0.57^**^/0.43^**^	0.04/0.20^*^	0.33^**^/0.29^**^
Open-mindedness	−0.04/−0.12	0.11/0.11	−0.07/−0.01	0.04/0.08	0.15/0.14	0.32^**^/0.22^*^	−0.05/0.09	−0.05/−0.07
Love of learning	0.13/0.00	0.22^*^/0.02	0.07/−0.06	0.16/0.03	0.15/0.05	0.28^**^/0.06	0.12/0.24^*^	0.13/−0.09
Perspective	0.04/−0.06	0.13/0.08	−0.10/−0.03	0.06/0.05	0.14/0.07	0.32^**^/0.20^*^	0.08/0.01	0.06/−0.01
**(2) COURAGE**								
Authenticity	0.17/0.10	0.47^**^/0.35^**^	0.35^**^/0.25^**^	0.31^**^/0.26^**^	0.31^**^/0.26^**^	0.44^**^/0.37^**^	0.19/0.23^*^	0.20^*^/0.13
Bravery	0.02/−0.03	0.17/0.13	−0.05/0.04	0.20^*^/0.11	0.24^*^/0.04	0.30^**^/0.26^**^	0.06/0.08	0.01/−0.10
Persistence	0.15/0.17	0.43^**^/0.44^**^	0.28^**^/0.24^**^	0.31^**^/0.41^**^	0.37^**^/0.33^**^	0.40^**^/0.48^**^	0.18/0.28^**^	0.17/0.25^**^
Zest	0.51^**^/0.42^**^	0.56^**^/0.53^**^	0.42^**^/0.48^**^	0.57^**^/0.57^**^	0.50^**^/0.43^**^	0.46^**^/0.40^**^	−0.09/0.16	0.48^**^/0.33^**^
**(3) HUMANITY**								
Kindness	0.10/−0.04	0.40^**^/0.30^**^	0.35^**^/0.30^**^	0.24^*^/0.22^*^	0.19^*^/0.14	0.38^**^/0.28^**^	0.06/0.16	0.18/0.05
Love	0.41^**^/0.21^*^	0.38^**^/0.35^**^	0.31^**^/0.33^**^	0.24^*^/0.18	0.32^**^/0.35^**^	0.27^**^/0.29^**^	0.10/0.10	0.29^**^/0.19^*^
Social intelligence	0.17/0.16	0.34^**^/0.33^**^	0.22^*^/0.29^**^	0.24^*^/0.22^*^	0.25^**^/0.25^**^	0.38^**^/0.33^**^	0.01/0.03	0.10/0.15
**(4) JUSTICE**								
Fairness	0.22^*^/0.00	0.43^**^/0.27^**^	0.30^**^/0.25^*^	0.31^**^/0.21^*^	0.29^**^/0.17	0.43^**^/0.24^*^	0.12/0.17	0.24^*^/0.02
Leadership	0.15/−0.10	0.31^**^/0.25^**^	0.24^*^/0.23^*^	0.24^*^/0.21^*^	0.28^**^/0.15	0.29^**^/0.29^**^	0.05/0.07	0.06/−0.08
Teamwork	0.19^*^/−0.05	0.39^**^/0.28^**^	0.37^**^/0.38^**^	0.28^**^/0.26^**^	0.24^*^/0.07	0.27^**^/0.17	0.13/0.05	0.20^*^/0.08
**(5) TEMPERANCE**								
Forgiveness	0.18/0.06	0.20^*^/0.15	0.19^*^/0.13	0.21^*^/0.17^*^	0.10/0.08	0.09/0.06	0.07/0.23^*^	0.27^**^/0.13
Modesty	0.06/0.05	0.11/0.17	0.06/0.14	0.17/0.15	0.10/0.17	0.02/0.12	0.20^*^/0.16	0.13/0.08
Prudence	−0.01/−0.03	0.15/0.24^*^	0.06/0.17	0.09/0.11	0.13/0.22^*^	0.16/0.25^**^	0.08/0.15	0.10/0.10
Self-regulation	0.17/0.16	0.34^**^/0.37^**^	0.28^**^/0.28^**^	0.30^**^/0.30^**^	0.26^**^/0.36^**^	0.26^**^/0.32^**^	0.15/0.22^*^	0.14/0.19^*^
**(6) TRANSCENDENCE**								
Appreciation of beauty/excellence	0.17/−0.03	0.17/0.05	0.11/0.06	0.17/0.11	0.15/0.04	0.15/0.03	0.08/0.05	0.14/−0.09
Gratitude	0.45^**^/0.27^**^	0.48^**^/0.41^**^	0.34^**^/0.34^**^	0.39^**^/0.41^**^	0.47^**^/0.38^**^	0.32^**^/0.35^**^	0.18/0.14	0.42^**^/0.17
Hope	0.67^**^/0.41^**^	0.59^**^/0.48^**^	0.42^**^/0.34^**^	0.30^**^/0.32^**^	0.63^**^/0.45^**^	0.40^**^/0.39^**^	0.22^*^/0.42^**^	0.66^**^/0.42^**^
Humor	0.30^**^/0.19^*^	0.42^**^/0.34^**^	0.37^**^/0.32^**^	0.41^**^/0.32^**^	0.23^*^/0.20^*^	0.36^**^/0.29^**^	−0.05/0.01	0.27^**^/0.20^*^
Spirituality	0.28^**^/0.31^**^	0.23^*^/0.36^**^	0.25^**^/0.34^**^	0.24^*^/0.39^**^	0.29^*^/0.27^**^	0.01/0.24^*^	−0.04/0.12	0.32^**^/0.30^**^
VIA-IS total score	0.41^**^/0.20^*^	0.63^**^/0.53^**^	0.42^**^/0.42^**^	0.51^**^/0.47^**^	0.52^**^/0.41^**^	0.58^**^/0.50^**^	0.16/0.27^**^	0.41^**^/0.21^*^

*N = 117; controlled for sex, age, and family; t1/t2, values at t1 and t2; partial correlation coefficients (two tailed): correlations of < 0.10, none, 0.10–0.29, weak, 0.30–0.49, moderate, ≥50 = high*,

* *statistical significance level of p = < 0.05*,

** *statistical significance level of p < 0.01; SWB, subjective well-being; PWB, psychological well-being; strengths measured by the Values in Action Inventory-120; well-being measured by the Comprehensive Inventory of Thriving*.

### Which character strengths are most strongly associated with PWB?

We had hypothesized that the happiness strengths at t1 would be correlated most strongly with PWB at t1 or t2 (hypothesis 2). Results confirmed hypothesis 2. High to moderate correlations were found for hope (*r* = 0.59/0.48), zest (*r* = 0.56/0.53), gratitude (*r* = 0.48/0.41), curiosity (*r* = 0.52/0.48) and love (*r* = 0.38/0.35). Compared to the correlations of character strengths and SWB, a greater number of character strengths was also moderately correlated with PWB at t1 and t2 (Table 2). No other character strength was significantly higher correlated with PWB than love (*p* > 0.05).

### Does “good character” in general have a stronger association with PWB than with SWB?

We had hypothesized that the “good character” (as measured by the VIA-120 total score) at t1 would show a significantly higher correlation with PWB than with SWB at t1 and t2 (hypothesis 3). This was confirmed by significantly higher correlation coefficients (*p* < 0.001) for PWB (*r* = 0.63/0.53) than SWB (*r* = 0.41/0.20). In particular, the PWB subcategories of mastery, meaning and engagement (large effect sizes), followed by relationships and optimism (moderate effect sizes), were significantly correlated with strengths possession in general, whereas autonomy showed a weak but still a significant relationship. This PWB aspect was by far the one with the lowest correlation with character strengths in general (Table 2).

### How are the character strengths related to psychological well-being aspects?

Higher scores on measures of the 24 character strengths (t1) were associated with higher scores concerning the six aspects of PWB at t1 and t2 when controlling for demographics (strengths are given in an ascending order and have moderate partial correlations at least; Table 2). We performed stepwise multiple regression analyses for all character strengths that were previously identified as significantly correlated at each respective time. The aim was to identify the character strengths that would explain the most unique variance (Table 3). The following focuses on results at t2; all results can be found in Table 3.

**Table 3:** Regression coefficients of character strengths entered in stepwise regression analyses to predict the different aspects of PWB at t1 and t2.

**Character strengths at t1**	**Relationships t1/t2**	**Engagement t1/t2**	**Meaning t1/t2**	**Mastery t1/t2**	**Autonomy t1/t2**	**Optimism t1/t2**
**(1) WISDOM AND KNOWLEDGE**						
Creativity				0.18^*^/–		
Curiosity				0.34^***^/0.25^**^		
Open-mindedness						
Love of learning						
Perspective						
**(2) COURAGE**						
Authenticity	0.18^*^/–			0.22^**^/–		
Bravery						
Persistence		0.19^*^/0.27^***^	0.17^*^/–	–/0.39^***^		
Zest	0.21^*^/0.34^***^	0.51^***^/0.42^***^	0.26^**^/0.23^**^			0.25^**^/–
**(3) HUMANITY**						
Kindness						
Love				–/0.20^*^		
Social intelligence				0.16^*^/–		
**(4) JUSTICE**						
Fairness						
Leadership						
Teamwork	0.21^*^/0.27^**^					
**(5) TEMPERANCE**						
Forgiveness						
Modesty						
Prudence						
Self-regulation			–/0.23^**^			
**(6) TRANSCENDENCE**						
**Appreciation of beauty/excellence**						
Gratitude						
Hope	0.24^**^/–		0.48^***^/0.30^**^	0.17^*^/–	0.22^*^/0.42^***^	0.55^***^/0.37^***^
Humor						
Spirituality	–/0.27^**^	–/0.24^**^				–/0.19^*^
*R^2^*	0.31/0.31	0.36/0.42	0.47/0.29	0.47/0.31	0.03/0.19	0.48/0.21

N = 117; ^#^only the variables identified as significantly correlated in partial correlation were included; controlled for sex, age and family; values are given for only the variables identified as moderately or highly correlated with the respective aspect of psychological well-being at t1 or t2; R^2^, corrected variance explained by demographics and character strengths;

* *p < 0.05*,

** *p < 0.01*,

*** *p < 0.001; t1/ t2, values at t1 and t2; strengths measured by the Values in Action Inventory-120; well-being measured by Comprehensive Inventory of Thriving*.

*Relationships* were moderately related to a broad range of strengths (step 1, Table 2). We found zest, teamwork and spirituality to be the most relevant strengths for this aspect of PWB (step 2, Table 3).

*Engagement* was also moderately related to a broad spectrum of strengths. We found high effects for zest and curiosity. Regression analyses identified zest as the most important character strength, followed by persistence and spirituality.

For *meaning*, we found several moderate effects. Hope, zest and self-regulation were shown to be the most important character strengths by stepwise regression.

*Mastery* had the highest amount of significantly correlated character strengths (19 and 18 strengths at t1 and t2, respectively). Furthermore, compared with the other PWB aspects, mastery was most strongly influenced by the wisdom and knowledge strengths (Table 2). We found the highest standardized regression coefficients for persistence, followed by curiosity and love.

For *autonomy*, hope was the only strength with a moderate effect.

For *optimism*, the character strengths of hope and spirituality were the most important strengths.

We found some differences between t1 and t2 (Table 3): Besides the most important strengths of t2 (zest, teamwork and spirituality), the PWB dimension of relationships was additionally associated with hope and authenticity. Meaning at t1 was also significantly related to hope, zest and as well as to persistence, but not to self-regulation. Optimism was not only related to hope, but also to zest at t1. Relationships, engagement and meaning were associated with spirituality only at t2, but not in the cross-sectional design.

## General discussion

This study was the first to examine different relationships between the 24 character strengths and specific well-being aspects (SWB and six aspects of PWB) in a sample of medical students at two measurement times. Former studies had primarily focused on SWB (e.g., Park et al., 2004; Ruch et al., 2007). Harzer and Ruch (2015) emphasized the importance of “much more fine-grained investigations” (p. 11) to get more information about relationships between character strengths and a broad range of relevant variables, such as different well-being aspects. Based on this, we analyzed the specific relationships between the character strengths, SWB and six aspects of PWB, and further identified the most relevant strengths at two measurement times.

Firstly, results showed that the general possession of character strengths was more strongly associated with PWB than with SWB. This shows in a first step that there are essential differences between these two well-being aspects. Secondly, similar to previous studies (e.g., Park et al., 2004; Peterson et al., 2005, 2007) we found the so-called happiness strengths of hope, zest, gratitude, curiosity and love to be central to SWB and PWB aspects. While we found SWB and PWB to be at least moderately related to hope, zest and curiosity, PWB was strongly correlated to a broader range of character strengths (Table 2). Our results confirmed that there is a bigger picture of well-being in connection with character strengths to explore. As a next step, six aspects of PWB were analyzed separately. We found that meaning, autonomy and optimism were all linked most strongly to hope. Relationships and engagement were most related to zest. Mastery was the PWB aspect which had the greatest amount of significant correlations with various character strengths. More than half of the character strengths had at least moderate partial correlations with mastery. Moreover, mastery was the only dimension which was considerably influenced by the wisdom and knowledge strengths. Persistence showed the strongest relationship with mastery at t2. These findings suggest that a broad range of character strengths may provide the opportunity to positively influence the subjective sense of being competent in managing daily tasks or challenges. Research may address the question whether these associations are attributable to a direct influence, or if mastery is more of a by-product of actively using one's strengths. Autonomy was by far the PWB dimension with the lowest correlation with character strengths in general (no correlation at t1, moderate correlation at t2). The intercorrelation between autonomy at t1 and t2 was also significantly lower than those of other well-being aspects (Table 1). At the level of the individual, there was a tendency toward higher autonomy at t2 compared with t1, which may be a reason for the discrepancy between t1 and t2. Perhaps the medical students explored more possibilities to be autonomous in a broader range of settings by using their character strengths (e.g., hope) after 1 year of studying. Despite the lower correlation between autonomy and strengths possession, a Beta of 0.42 warrant further exploration of the potential of the strength of hope in particular to promote autonomy. Results for creativity, bravery, fairness, and prudence differed between t1 and t2 (Table 2). Creativity, bravery, and fairness showed higher correlation coefficients at t1. Future studies may analyze if these strengths are causally related. If this is the case a possible explanation may be that these strengths display their positive impact on PWB more directly and in the short term, whereas the positive effects of prudence only become relevant at t2.

Our study results showed hope to be the most important character strength for most of the PWB aspects. Proyer et al. (2011) found hope and spirituality to be the best predictors of future life satisfaction (as one aspect of SWB). In our study, spirituality was not strongly related to the well-being outcomes (both SWB and PWB) at t1. Spirituality is defined as “having coherent beliefs about the higher purpose and meaning of the universe […] that shape conduct and provide comfort” (VIA Institute of Character, 2016). This shows that spirituality is something long-lasting, which might become of importance only over time. Appreciation of beauty and excellence, open-mindedness, modesty, perspective, love of learning, creativity, bravery, and prudence were the character strengths with the smallest effects on most of the well-being aspects. These results are similar to those reported by Park et al. (2004), who found appreciation of beauty and excellence, open-mindedness, modesty, love of learning and creativity to be the five character strengths least related to life satisfaction. The novelty of our study in this case is the extension of these results to PWB aspects as well.

As far as we know, there have been no previous studies that investigated these links in a sample of medical students. However, research has shown that medical students are especially at risk of experiencing burnout and lower levels of well-being throughout their education (e.g., Dyrbye et al., 2008, 2010; Dyrbye and Shanafelt, 2011). These studies indicate the need for learning more about possible interventions that can be implemented in medical curricula to proactively prevent burnout and counteract low levels of well-being. Character strengths are important, but unfortunately often neglected, resources for organizations in general (Peterson and Park, 2006). Fostering knowledge and offering opportunities to use one's character strengths in education and work contexts may positively influence well-being (Peterson and Park, 2006) and health (Veenhoven, 2008).

### Theoretical and practical implications

The study offers some theoretical implications. The happiness strengths were highly correlated with SWB and a broad range of PWB aspects. In general, these strong relationships mirror the assumption that tonic strengths fit in more situations than phasic strengths: Strengths that are relevant in a broader range of situations are called tonic (e.g., gratitude or zest), while phasic strengths are relevant for specific situations (e.g., bravery or leadership) (Peterson and Seligman, 2004). The VIA-120 measures the frequency of behavior patterns and tonic strengths could be generally used more often in a broader range of situations and may therefore be more central. Another reason for the high correlation between happiness strengths and especially SWB is that character strengths have something in common with positive emotions (Fredrickson, 2009). Güsewell and Ruch (2012) analyzed the relationship of character strengths and dispositional positive emotions in a German-speaking sample. They found low to moderate correlations between character strengths and emotions in general, but moderate to high correlations of a few strengths–emotions pairs. The highest correlations for the five happiness strengths in their study were: curiosity and hope with contentment (0.51^**^; 0.67^**^), zest and gratitude with joy (0.63^**^; 0.54^**^) and love (as a strength) with love (as an emotion) (0.53^**^). The overlap between these pairings and results of longitudinal studies (e.g., Proyer et al., 2011) show the potential of positively influencing (subjective) well-being through character strengths interventions.

Our results also have several practical implications: The idea of improving the well-being of medical students by using character strengths interventions, in particular by fostering the character strengths of hope, zest, gratitude, curiosity and love based on our results warrant future research. Furthermore, if a specific well-being aspect should be targeted it may be helpful to choose a character strengths intervention that is aimed at a strength which is especially related to this well-being aspect. For example, Gander et al. (2016) adapted the well-known exercise “three good things”, where participants have to write down three positive things of the day and reflect what they have contributed to let them happen (Seligman et al., 2005), to different well-being aspects. The authors put the focus of the exercise in relation to one of the well-being aspects pleasure, engagement, meaning, positive relationships or accomplishment. To find a suitable and potentially effective intervention for the respective well-being aspect which should be addressed, knowing about the basic relationships between well-being aspects and character strengths is an important resource. This study is the first to report results of relationships between specific characters strengths and a broad range of well-being aspects in a sample of medical students of an Austrian university. The study shows that the PWB aspects *mastery* and *relationships* both were relevantly linked to a great amount of character strengths, indicating that these aspects of well-being may broadly benefit from different character strengths interventions. Across all results hope, zest and spirituality emerged as important key variables for a broad range of well-being aspects at t2. This provides sufficient rational to support future research in this area focusing on developing interventions that target these strengths in particular. For example, experimental research fostering the application of hope as a character strength could demonstrate whether well-being can be influenced directly or not. An example of targeting hope as a character strength is the exercise “best possible self,” where people write for 20 min about a positive possible self in the future, imagining the best that could happen in this period of time (King, 2001; Layous et al., 2013). Additionally, assuming a direct effect of curiosity and gratitude on hope and zest, it is plausible that fostering curiosity or gratitude may be an indirect way to foster hope and zest as well, because of the high intercorrelations between curiosity and both zest and hope, and gratitude and zest/hope. Causal links may be examined in future research.

To summarize, our study offers a fine-grained analysis of the relation between specific character strengths and a broad range of well-being aspects. Our analyses have shown that specific character strengths are linked in distinct ways to SWB and different aspects of PWB in medical students. The six subscales of PWB varied substantially in their associations with the 24 character strengths. These results underline the importance of more precise differentiation in research on character strengths and well-being. There is a need to investigate potential underlying causal relationships, which may help plan and design interventions to address the important research question of how to increase different well-being aspects by promoting specific character strengths. Future analyses may also inform practice with a view to targeting specific well-being aspects through interventions that focus on one or more corresponding character strengths. For example, in order to enhance the PWB aspects of autonomy (or meaning), a character strengths intervention targeting hope (such as the exercise “best possible self”) may be particularly beneficial. This further may allow for a more focused application of positive psychological strengths interventions in medical education.

### Limitations and further research

The study was faced with several limitations:

Firstly, the sample is limited in its generalizability by the fact that all medical students came from one medical university in Austria, and by the relatively small sample size due to the study design (two times of measurement) and the length of the applied questionnaires. Moreover, all data was self-reported, which may have led to inflated correlations (common method bias). However, according to Podsakoff et al. (2003), separating measurements over time is one way of attenuating the risk of common method bias. In order to attain more generalizable results, future research should replicate these findings at different stages over the medical career (including more measurement points or longer intervals in between), in different countries (to take potential cultural differences into account; Joshanloo, 2014) and additionally, in the general population or in other specific samples over time. The impact of culture on specific links between character strengths and well-being has yet not been sufficiently empirically addressed in the scientific literature. Potential differences regarding the well-being of medical students living in western vs. non-western countries (e.g., with respect to the definition of the self, the “standing” of hedonism vs. euidaimonism, accepting vs. avoiding negative emotions, control/mastery vs. interpersonal harmony, life satisfaction vs. contentment, relevance of spirituality and religion; Joshanloo, 2014) and developmental aspects (students vs. physicians) should be addressed in future cross-cultural studies.

Nevertheless, in spite of the specificity of the sample, our study replicated the character strengths previously shown to be the most relevant to well-being (hope, zest, gratitude, curiosity, and love) in general populations (Park et al., 2004; Peterson et al., 2005, 2007). This shows that results from samples of the general population also have relevance for medical students in particular. Knowing which character strengths serve as protective factors and positive predictors of SWB and PWB may be helpful in shaping medical education with a view to fostering the well-being of students and mitigating risks such as burnout.

Secondly, the general question of causal vs. indicator variables is a common issue in well-being research (Fayers and Hand, 2002). The measures of well-being and character strengths can include variables that are indicators of the magnitude of the underlying construct, but they can also include variables that are part of the definition of the construct. That people with higher well-being also show higher values in specific character strengths (or higher means in general) does not automatically imply that these strengths lead to well-being in a causal sense. Perhaps well-being is not the consequence of character strengths, but reflects aspects of character strengths (or vice versa).

Thirdly, another limitation lies in the partly relatively low reliability coefficients of strengths scores. In contrast to previous validations of the VIA-IS and the VIA-120, some of the Cronbach's alphas in our study were lower than expected: Love of learning (t1/t2), authenticity (t1), love (t1), social intelligence (t1/t2), leadership (t1), teamwork (t2), forgiveness (t1/t2), prudence (t2), self-regulation (t1/t2) and appreciation of beauty (t2) had alphas ranging between 0.6 and 0.7, which indicates a questionable reliability in slight contrast to other studies (e.g., Ruch et al., 2010; Littman-Ovadia, 2015). In particular, modesty had an alpha of 0.57 at t1, which casts doubt on the internal consistency of that subscale. Means of modesty were relatively low and highly correlated between t1 and t2. Modesty showed no relevant relationships with any of the well-being aspects. Due to the low reliability, however, these particular results should be interpreted with caution.

The exclusion of the traditional clustering of character strengths into six “virtues” in our statistical analyses is an impulse for further thought rather than a limitation. Several studies have conducted factor analyses before but different factor structures were found: Three factors (McGrath, 2015), five (e.g., Peterson et al., 2008; Ruch et al., 2010; Harzer and Ruch, 2014) or six factors (Ruch and Proyer, 2015) were identified through statistical analyses. In addition to the lack of empirical evidence for six virtues based on the VIA-IS or VIA-120, all current interventions address character strengths, not virtues as an empirical construct. An analysis at the level of strengths allows for an interpretation of the effect of each character strength on well-being. Therefore we decided to use the theoretical construct of virtues only as conceptual clusters.

In this study we focused on distinct relationships between the different character strengths and several aspects of well-being. Another focus would have been an analysis based on participants' signature strengths, which have been shown to be especially relevant for the individual (e.g., for work satisfaction; Harzer and Ruch, 2012). Future research should address the level of character strengths (e.g., highest strength scores vs. lowest strengths scores) in a longitudinal study design in relation to well-being. Despite this issue, the fact that situational factors may allow or prohibit the application of strengths remains completely unaddressed in this analysis, and warrants further exploration.

Based on the results of this study, it is worth investigating the potential of character strengths interventions in medical education in order to understand potential underlying causal relationships. In practice, a character strengths approach may proactively improve students' well-being. Future research might address whether improvements in medical curricula can be achieved by (a) identifying character strengths that are generally helpful to medical students for specific outcomes (e.g., accomplishment, academic success, work engagement, or burnout), (b) investigating the effects of becoming aware of one's (signature) strengths, (c) assessing the impact of fostering these strengths through shaping studying conditions accordingly, and (d) evaluating the teaching and promotion of individual character strengths (e.g., through activities that build character strengths) in the educational context.

## Conclusions

In conclusion, the present study has the following implications:

Firstly, our fine-grained analyses created first knowledge about different relational aspects of character strengths and specific well-being aspects. Secondly, the results have shown that specific character strengths are linked in distinct ways to SWB and different aspects of PWB in medical students. This indicates that it is necessary for future research to take up a detailed strategy when exploring research questions in the field of character strengths and well-being. Thirdly, based on this study we recommend the use of a clear and precise wording in studies operationalizing (different) aspects of well-being. Finally, our findings suggest that it is worth investigating the potential of character strengths interventions in medical education in order to understand potential underlying causal relationships. Future research may show if improvements of the medical curricula can be achieved by including the concept of character strengths in order to foster the well-being of medical students.

## Ethics statement

This study was carried out in accordance with the recommendations of “The Board for Ethical Questions in Science of the University of Innsbruck” with written informed consent from all subjects. All subjects gave written informed consent in accordance with the Declaration of Helsinki. The protocol was approved by the “The Board for Ethical Questions in Science of the University of Innsbruck.”

## Author contributions

All authors were substantially involved in the planning of the study, the interpretation of data and revision of the article. MH conducted the data collection, data analysis and the writing of the article.

### Conflict of interest statement

The authors declare that the research was conducted in the absence of any commercial or financial relationships that could be construed as a potential conflict of interest.

## References

[B1] Allan B. A. Duffy R. D. (2014). Examining moderators of signature strengths use and well-being: Calling and signature strengths level. J. Happiness Stud. 15, 323–337. 10.1007/s10902-013-9424-0

[B2] Brazeau C. M. L. R. Shanafelt T. Durning S. J. Massie F. S. Eacker A. Moutier C.. (2014). Distress among matriculating medical students relative to the general population. Acad. Med. J. Assoc. Am. Med. Coll. 89, 1520–1525. 10.1097/ACM.0000000000000482 25250752

[B3] Buschor C. Proyer R. T. Ruch W. (2013). Self- and peer-rated character strengths: how do they relate to satisfaction with life and orientations to happiness? J. Posit. Psychol. 8, 116–127. 10.1080/17439760.2012.758305

[B4] Deci E. L. Ryan R. M. (2008). Hedonia, eudaimonia, and well-being: an introduction. J. Happiness Stud. 9, 1–11. 10.1007/s10902-006-9018-1

[B5] Diener E. Wirtz D. Biswas-Diener R. Tov W. Kim-Prieto C. Choi D.-w. (2009). New measures of well-being, in Assessing Well-being: The Collected Works of Ed Diener, ed Diener E. (New York, NY: Springer Science + Business Media), 247–266.

[B6] Douglass R. P. Duffy R. D. (2014). Strengths use and life satisfaction: a moderated mediation approach. J. Happiness Stud. 16, 619–632. 10.1007/s10902-014-9525-4

[B7] Dyrbye L. N. Shanafelt T. D. (2011). Commentary: medical student distress: a call to action. Acad. Med. 86, 801–803. 10.1097/ACM.0b013e31821da481 21715992

[B8] Dyrbye L. N. Thomas M. R. Massie F. S. Power D. V. Eacker A. Harper W.. (2008). Burnout and Suicidal Ideation among U.S. Medical Students. Ann. Int. Med. 149, 334–W370. 10.7326/0003-4819-149-5-200809020-00008 18765703

[B9] Dyrbye L. N. Thomas M. R. Power D. V. Durning S. Moutier C. Massie F. S. Jr.. (2010). Burnout and serious thoughts of dropping out of medical school: a multi-institutional study. Acad. Med. J. Assoc. Am. Med. Coll. 85, 94–102. 10.1097/ACM.0b013e3181c46aad 20042833

[B10] Dyrbye L. N. Varkey P. Boone S. L. Satele D. V. Sloan J. A. Shanafelt T. D.. (2013). Physician satisfaction and burnout at different career stages. Mayo Clinic Proc. 88, 1358–1367. 10.1016/j.mayocp.2013.07.016 24290109

[B11] Dyrbye L. N. West C. P. Satele D. Boone S. Tan L. Sloan J.. (2014). Burnout among U.S. medical students, residents, and early career physicians relative to the general U.S. population. Acad. Med. 89, 443–451. 10.1097/ACM.0000000000000134 24448053

[B12] Fayers P. M. Hand D. J. (2002). Causal variables, indicator variables and measurement scales: an example from quality of life. J. R. Stat. Soc. Ser. A. 165, 233–253. 10.1111/1467-985X.02020

[B13] Fredrickson B. L. (2009). Positivity: Groundbreaking Research Reveals How to Embrace the Hidden Strength of Positive Emotions, Overcome Negativity, and Thrive. New York, NY: Crown Publishers/Random House.

[B14] Gander F. Proyer R. T. Ruch W. (2016). Positive psychology interventions addressing pleasure, engagement, meaning, positive relationships, and accomplishment increase well-being and ameliorate depressive symptoms: a randomized, placebo-controlled online study. Front. Psychol. 7:686. 10.3389/fpsyg.2016.00686 27242600 PMC4873493

[B15] Güsewell A. Ruch W. (2012). Are only emotional strengths emotional? Character strengths disposition to positive emotions. Appl. Psychol. Health Well Being 4, 218–239. 10.1111/j.1758-0854.2012.01070.x 26286979

[B16] Harzer C. Ruch W. (2012). When the job is a calling: the role of applying one's signature strengths at work. J. Posit. Psychol. 7, 362–371. 10.1080/17439760.2012.702784

[B17] Harzer C. Ruch W. (2014). The role of character strengths for task performance, job dedication, interpersonal facilitation, and organizational support. Hum. Perform. 27, 183–205. 10.1080/08959285.2014.913592

[B18] Harzer C. Ruch W. (2015). The relationships of character strengths with coping, work-related stress, and job satisfaction. Front. Psychol. 6:165. 10.3389/fpsyg.2015.00165 25767452 PMC4341515

[B19] Hausler M. Huber A. Strecker C. Brenner M. Höge T. Höfer S. (2017). Validation of a holistic measure for the construct of well-being - The German version of the Comprehensive Inventory of Thriving (CIT) and the short version Brief Inventory of Thriving (BIT). Diagnostica 63, 219–228. 10.1026/0012-1924/a000174

[B20] Institute on Character V. (2014). VIA Survey Psychometric Data [Internet]. Available online at: https://www.viacharacter.org/www/Research/Psychometric-Data

[B21] Joshanloo M. (2014). Eastern conceptualizations of happiness: fundamental differences in Eastern and Western views. J. Happiness Stud. 15, 475–493. 10.1007/s10902-013-9431-1

[B22] Judge T. A. Erez A. Bono J. E. Thoresen C. J. (2003). The core self-evaluatons scale: development of a measure. Person. Psychology 56, 303–331. 10.1111/j.1744-6570.2003.tb00152.x

[B23] Keyes C. L. M. Shmotkin D. Ryff C. D. (2002). Optimizing well-being: the empirical encounter of two traditions. J. Pers. Soc. Psychol. 82, 1007–1022. 10.1037/0022-3514.82.6.1007 12051575

[B24] King L. A. (2001). The health benefits of writing about life goals. Person. Soc. Psychol. Bull. 27, 798–807. 10.1177/0146167201277003

[B25] Layous K. Nelson S. K. Lyubomirsky S. (2013). What is the optimal way to deliver a positive activity intervention? The case of writing about one's best possible selves. J. Happiness Stud. 14, 635–654. 10.1007/s10902-012-9346-2

[B26] Littman-Ovadia H. (2015). Brief report: short form of the VIA inventory of strengths - construction and initial tests of reliability and validity. Int. J. Human. Soc. Sci. Educ. 2, 229–237. Available online at: https://www.arcjournals.org/pdfs/ijhsse/v2-i4/27.pdf

[B27] Littman-Ovadia H. Lavy S. Boiman-Meshita M. (2017). When theory and research collide: examining correlates of signature-strengths use at work. J. Happiness Stud. 18, 527–548. 10.1007/s10902-016-9739-8

[B28] Martínez-Martí M. L. Ruch W. (2014). Character strengths and well-being across the life span: data from a representative sample of German-speaking adults living in Switzerland. Front. Psychol. 5:1253. 10.3389/fpsyg.2014.01253 25408678 PMC4219388

[B29] McGrath R. E. (2015). Integrating psychological and cultural perspectives on virtue: the hierarchical structure of character strengths. J. Posit. Psychol. 10, 407–424. 10.1080/17439760.2014.994222

[B30] Park N. Peterson C. Seligman M. P. (2004). Strengths of character and well-being. J. Soc. Clin. Psychol. 23, 603–619. 10.1521/jscp.23.5.603.50748

[B31] Pearlin L. I. Schooler C. (1978). The structure of coping. J. Health Soc. Behav. 19, 2–21. 649936

[B32] Peterson C. Park N. (2006). Character strengths in organizations. J. Organ. Behav. 27, 1149–1154. 10.1002/job.398

[B33] Peterson C. Park N. (2011). Character strengths and virtues: their role in well-being, in Applied Positive Psychology: Improving Everyday Life, Health, Schools, Work, and Society, eds Donaldson S. I. Csikszentmihalyi M. Nakamura J. (New York, NY: Routledge/Taylor and Francis Group), 49–62.

[B34] Peterson C. Park N. Pole N. D'Andrea W. Seligman M. E. P. (2008). Strengths of character and posttraumatic growth. J. Trauma. Stress 21, 214–217. 10.1002/jts.20332 18404632

[B35] Peterson C. Park N. Seligman M. E. P. (2005). Orientations to happiness and life satisfaction: the full life versus the empty life. J. Happiness Stud. 6, 25–41. 10.1007/s10902-004-1278-z

[B36] Peterson C. Ruch W. Beermann U. Park N. Seligman M. E. P. (2007). Strengths of character, orientations to happiness, and life satisfaction. J. Posit. Psychol. 2, 149–156. 10.1080/17439760701228938

[B37] Peterson C. Seligman M. E. P. (2004). Character Strengths and Virtues: A Handbook and Classification. Washington, DC; New York, NY: Oxford University Press.

[B38] Podsakoff P. M. MacKenzie S. B. Lee J.-Y. Podsakoff N. P. (2003). Common method biases in behavioral research: a critical review of the literature and recommended remedies. J. Appl. Psychol. 88, 879–903. 10.1037/0021-9010.88.5.879 14516251

[B39] Proyer R. T. Gander F. Wyss T. Ruch W. (2011). The relation of character strengths to past, present, and future life satisfaction among German-speaking women. Appl. Psychol. Health Well Being 3, 370–384. 10.1111/j.1758-0854.2011.01060.x

[B40] Proyer R. T. Ruch W. Buschor C. (2013). Testing strengths-based interventions: a preliminary study on the effectiveness of a program targeting curiosity, gratitude, hope, humor, and zest for enhancing life satisfaction. J. Happiness Stud. 14, 275–292. 10.1007/s10902-012-9331-9

[B41] Quinlan D. Swain N. Vella-Brodrick D. A. (2012). Character strengths interventions: building on what we know for improved outcomes. J. Happiness Stud. 13, 1145–1163. 10.1007/s10902-011-9311-5

[B42] Ring L. Höfer S. McGee H. Hickey A. O'Boyle C. (2007). Individual quality of life: can it be accounted for by psychological or subjective well-being? Soc. Indic. Res. 82, 443–461. 10.1007/s11205-006-9041-y

[B43] Ruch W. Huber A. Beermann U. Proyer R. T. (2007). Character strengths as predictors of the “good life” in Austria, Germany and Switzerland, in Romanian Academy, ‘George Barit’ Institute of History, Department of Social Research; Studies and Researches in Social Sciences (Cluj-Napoca: Argonaut Press), 123–131.

[B44] Ruch W. Proyer R. T. (2015). Mapping strengths into virtues: The relation of the 24 VIA-strengths to six ubiquitous virtues. Front. Psychol. 6:460. 10.3389/fpsyg.2015.00460 25954222 PMC4404979

[B45] Ruch W. Proyer R. T. Harzer C. Park N. Peterson C. Seligman M. E. P. (2010). Values in Action Inventory of Strengths (VIA-IS): Adaptation and validation of the German version and the development of a peer-rating form. J. Individ. Differ. 31, 138–149. 10.1027/1614-0001/a000022

[B46] Ryan R. M. Deci E. L. (2000). Self-determination theory and the facilitation of intrinsic motivation, social development, and well-being. Am. Psychol. 55, 68–78. 10.1037/0003-066X.55.1.68 11392867

[B47] Ryff C. D. (1995). Psychological well-being in adult life. Curr. Dir. Psychol. Sci. 4, 99–104.

[B48] Scheier M. F. Carver C. S. (1985). Optimism, coping, and health: assessment and implications of generalized outcome expectancies. Health Psychol. 4, 219–247. 4029106 10.1037//0278-6133.4.3.219

[B49] Seligman M. (2002). Authentic Happiness: Using the New Positive Psychology to Realize Your Potential for Lasting Fulfillment. New York, NY: Free Press.

[B50] Seligman M. (2011). Flourish: A Visionary New understanding of Happiness and Well-being. New York, NY: Free Press.

[B51] Seligman M. Csikszentmihalyi M. (2000). Positive psychology: an introduction. Am. Psychol. 55, 5–14. 10.1037/0003-066X.55.1.5 11392865

[B52] Seligman M. Steen T. A. Park N. Peterson C. (2005). Positive psychology progress: empirical validation of interventions. Am. Psychol. 60, 410–421. 10.1037/0003-066X.60.5.410 16045394

[B53] Shoshani A. Slone M. (2013). Middle school transition from the strengths perspective: young adolescents' character strengths, subjective well-being, and school adjustment. J. Happiness Stud. 14, 1163–1181. 10.1007/s10902-012-9374-y

[B54] Steiger J. H. (1980). Tests for comparing elements of a correlation matrix. Psychol. Bull. 87, 245–251. 10.1037//0033-2909.87.2.245

[B55] Su R. Tay L. Diener E. (2014). The development and validation of the comprehensive inventory of thriving (CIT) and the brief inventory of thriving (BIT). Appl. Psychol. Health Well Being 6, 251–279. 10.1111/aphw.12027 24919454

[B56] Veenhoven R. (2008). Healthy happiness: effects of happiness on physical health and the consequences for preventive health care. J. Happiness Stud. 9, 449–469. 10.1007/s10902-006-9042-1

[B57] VIA Institute of Character (2016). VIA Classification of Character Strengths [Internet]. Available online at: http://www.viacharacter.org/www/Character-Strengths/VIA-Classification

[B58] Voltmer E. Kötter T. Spahn C. (2012). Perceived medical school stress and the development of behavior and experience patterns in German medical students. Med. Teach. 34, 840–847. 10.3109/0142159X.2012.706339 22917267

[B59] Wagner L. Ruch W. (2015). Good character at school: Positive classroom behavior mediates the link between character strengths and school achievement. Front. Psychol. 6:610. 10.3389/fpsyg.2015.00610 26029144 PMC4432234

[B60] Weber M. Ruch W. Littman-Ovadia H. Lavy S. Gai O. (2013). Relationships among higher-order strengths factors, subjective well-being, and general self-efficacy - The case of Israeli adolescents. Pers. Individ. Dif. 55, 322–327. 10.1016/j.paid.2013.03.006

