# Supplementary material for: Distinguishing Relational Aspects of Character Strengths with Subjective and Psychological Well-being
Source: Front Psychol. 2017 Jul 11;8:1159. doi: 10.3389/fpsyg.2017.01159 (PMC5504157; doi:10.3389/fpsyg.2017.01159)
Supplement: Supplementary file 1 [file Table1.pdf]

Supplementary Material

Distinguishing Relational Aspects of Character Strengths with Subjective and Psychological Well-being

Melanie Hausler*, Cornelia Strecker, Alexandra Huber, Mirjam Brenner, Thomas Höge, Stefan Höfer

*** Correspondence:** Melanie Hausler: melanie.hausler@i-med.ac.at

Supplementary Table 1: Character strengths overview[#]

**(1) Wisdom and knowledge: cognitive strengths that entail the acquisition and use of knowledge**	
Creativity	Thinking of novel and productive ways to conceptualize and do things.
Curiosity	Taking an interest in ongoing experience for its own sake; finding things fascinating; exploring and discovering.
Open-mindedness/judgment	Thinking things through and examining them from all sides; being able to change one's mind in light of evidence.
Love of learning	Mastering new skills, topics, and bodies of knowledge.
Perspective	Being able to provide wise counsel; having ways of looking at the world that makes sense.
**(2) Courage: Emotional strengths that involve the exercise of will to accomplish goals in the face opposition, external or internal.**	
Authenticity/honesty	Speaking the truth and presenting oneself in a genuine way; taking responsibility for one's feelings and actions.
Bravery	Not shrinking from threat, challenge, difficulty, or pain; speaking up for what is right; acting on convictions.
Persistence/perseverance	Finishing what one starts and finding pleasure in it.
Zest/enthusiasm	Approaching life with excitement and energy; feeling alive and activated.
**(3) Humanity: Interpersonal strengths that involve tending and befriending other**	
Kindness	Doing favors and good deeds for others; helping and taking care of them.

Love	Valuing close relations with others.
Social intelligence	Being aware of the motives and feelings of others and oneself; knowing what to do to fit into social situations.

(4) Justice: Civic strengths that underlie healthy community life

Fairness	Treating all people the same according to notions of fairness and justices.
Leadership	Encouraging group activities, seeing that they happen and meanwhile maintaining good relations within the group.
Teamwork	Working well as a group or team member; being loyal.

(5) Temperance: Strengths that protect against excess

Forgiveness	Forgiving those who have done wrong; not being vengeful.
Modesty/humility	Letting one's accomplishments speak for themselves.
Prudence	Being careful about one's choices; not saying or doing things that might later be regretted.
Self-regulation	Regulating what one feels and does; being disciplined.

(6) Transcendence: Strengths that forge connections to the larger universe and provide meaning

Appreciation of beauty and excellence	Noticing and appreciating beauty, excellence or skilled performance in various domains of life.
Gratitude	Being aware of and thankful for the good things that happen.
Hope	Expecting the best in the future and working to achieve it.
Humor	Liking to laugh and tease; bringing smiles to other people.
Spirituality/purpose	Having coherent beliefs about the higher purpose and meaning of the universe that shape conduct and provide comfort.

[#] adapted from: http://www.viacharacter.org/www/Character-Strengths/VIA-Classification and from: Peterson, C., & Seligman, M. E. P. (2004). Character strengths and virtues: A handbook and classification. New York: Oxford University Press and Washington, DC: American Psychological Association. www.viacharacter.org
